# P02.126. Addition of chiropractic manipulative therapy to standard medical care may improve outcomes for acute low back pain in active-duty military personnel

**DOI:** 10.1186/1472-6882-12-S1-P182

**Published:** 2012-06-12

**Authors:** C Goertz, C Long, M Hondras, R Petri, W Meeker, D Lawrence, E Owens

**Affiliations:** 1 Palmer Center for Chiropractic Research, Davenport, USA; 2 Fort Bliss Physical Medicine and Integrative Health Services , El Paso, USA; 3 Palmer College of Chiropractic, San Jose, USA; 4 Private Practitioner, Minneapolis, USA

## Purpose

The primary aim of this pragmatic clinical trial was to assess changes in pain levels and physical functioning in response to standard medical care (SMC) vs. SMC plus chiropractic manipulative therapy (CMT) for the treatment of low back pain (LBP) among 18 to 35-year-old active-duty military personnel based at Ft. Bliss, El Paso, TX.

## Methods

This was a prospective, 2-arm randomized controlled pilot study for participants with acute low back pain. SMC included education about self-management, analgesics and anti-inflammatory agents, physical therapy and modalities such as heat/ice. CMT included mobilization, brief massage, modalities such as heat/ice, exercises, advice for activities of daily living, postural/ergonomic advice and high-velocity low-amplitude spinal manipulation for up to 2 visits weekly for 4 weeks.

## Results

A total of 213 soldiers were screened and 91 were randomized to treatment groups in a 1:1 allocation ratio. The mean age of participants was 26 years; 86% were male and 63% were white. The median duration of their current LBP episode was 9 days and 43% had radicular signs. Mixed linear effects models were fit for each of the 3 outcomes over the week 2 and 4 endpoints, adjusting for the respective baseline level of the outcome. After 4 weeks of care, the SMC+CMT group had significantly more improvement than SMC alone in the Roland Morris disability scores [adjusted mean (95% CI) 4.0 (1.3, 6.7)]; the numerical rating scale for current pain [2.2 (1.2, 3.1)]; and the Back Pain Functional Scale [7.7 (2.6, 12.9)].

## Conclusion

This pragmatic pilot study provides preliminary data showing clinically and statistically significant differences in improvement of pain and physical functioning between treatment groups, favoring the SMC+CMT group for young, racially diverse adults with low back pain. A larger clinical trial has recently been funded by the Department of Defense to confirm these results.

